# Cognition-Mortality Associations Are More Pronounced When Estimated Jointly in Longitudinal and Time-to-Event Models

**DOI:** 10.3389/fpsyg.2021.708361

**Published:** 2021-08-06

**Authors:** Stephen Aichele, Sezen Cekic, Patrick Rabbitt, Paolo Ghisletta

**Affiliations:** ^1^Department of Human Development and Family Studies, Colorado State University, Fort Collins, CO, United States; ^2^Faculty of Psychology and Educational Sciences, University of Geneva, Geneva, Switzerland; ^3^Department of Experimental Psychology, University of Oxford, Oxford, United Kingdom; ^4^Swiss National Center of Competence in Research LIVES—Overcoming Vulnerability: Life Course Perspectives, Universities of Lausanne and of Geneva, Geneva, Switzerland; ^5^Swiss Distance University Institute, Brig, Switzerland

**Keywords:** cognitive decline, survival, lifespan, joint models, Bayesian, longitudinal

## Abstract

With aging populations worldwide, there is growing interest in links between cognitive decline and elevated mortality risk—and, by extension, analytic approaches to further clarify these associations. Toward this end, some researchers have compared cognitive trajectories of survivors vs. decedents while others have examined longitudinal changes in cognition as predictive of mortality risk. A two-stage modeling framework is typically used in this latter approach; however, several recent studies have used joint longitudinal-survival modeling (i.e., estimating longitudinal change in cognition conditionally on mortality risk, and vice versa). Methodological differences inherent to these approaches may influence estimates of cognitive decline and cognition-mortality associations. These effects may vary across cognitive domains insofar as changes in broad fluid and crystallized abilities are differentially sensitive to aging and mortality risk. We compared these analytic approaches as applied to data from a large-sample, repeated-measures study of older adults (*N* = 5,954; ages 50–87 years at assessment; 4,453 deceased at last census). Cognitive trajectories indicated worse performance in decedents and when estimated jointly with mortality risk, but this was attenuated after adjustment for health-related covariates. Better cognitive performance predicted lower mortality risk, and, importantly, cognition-mortality associations were more pronounced when estimated in joint models. Associations between mortality risk and crystallized abilities only emerged under joint estimation. This may have important implications for cognitive reserve, which posits that knowledge and skills considered well-preserved in later life (i.e., crystallized abilities) may compensate for declines in abilities more prone to neurodegeneration, such as recall memory and problem solving. Joint longitudinal-survival models thus appear to be important (and currently underutilized) for research in cognitive epidemiology.

## Introduction

The boundaries which divide Life from Death are at best shadowy and vague. Who shall say where the one ends, and where the other begins?

—Edgar Allen Poe, *The Premature Burial*

Cognitive abilities (e.g., verbal skill, abstract reasoning) decline at different rates across the adult lifespan and with differential sensitivity to biological and health-related influences (Baltes et al., 2006). Cognitive declines are also closely associated with elevated mortality risk, a relation that persists even after accounting for sociodemographic and health-related variables (Anstey et al., 2006; Aichele et al., 2016). Increased knowledge of cognition-mortality associations can inform strategies to support mental wellness in later adulthood, provide caregivers with insight about the scope and timeframe of end-of-life mental declines, and may be useful for diagnostic purposes: E.g., processing speed declines as indicative of elevated vascular risk (Batterham et al., 2012; Aichele et al., 2016). However, this knowledge hinges on methodological criteria.

To date, favored approaches for studying cognition-mortality associations have included comparing cognitive trajectories of survivors vs. decedents, modeling cognitive change as a function of time-to-death (i.e., terminal cognitive decline in samples of decedents), and the use of two-stage procedures for estimating survival times as a function of differences, and longitudinal changes, in cognitive performance. More recently, several studies have used joint longitudinal-survival models (Rizopoulos et al., 2014) to estimate, concurrently, intraindividual cognitive change conditionally on interindividual differences in mortality risk, and vice versa. This latter approach may provide increased statistical efficiency and lessen statistical bias (i.e., because longitudinal performance may inform death-related attrition, and mortality-related processes may influence longitudinal performance). In the current study, we compare outcomes from two-stage and joint procedures for modeling longitudinal cognition-survival associations. We first highlight considerations for estimating cognitive trajectories in the presence of death-related attrition. We then turn to cognitive ability as predictive of mortality risk (cognitive epidemiology), the focal application for the study.

### Cognitive Trajectories, Accounting for Differences in Mortality Risk

Adult lifespan psychology studies have typically sought to describe cognitive changes that are well characterized by chronological age (Hertzog and Nesselroade, 2003; Sliwinski et al., 2006). Outcomes from these studies have prompted researchers to distinguish between broad “crystallized” and “fluid” abilities. Crystallized intelligence (Gc) reflects accumulated knowledge (e.g., education, experience, acculturation) and is generally thought to remain more-or-less stable during adulthood. Fluid intelligence (Gf), which is linked to basic information-processing efficiency, is more sensitive to biological and health-related influences and therefore declines more, and with greater variability, across the lifespan (Horn and Cattell, 1966, 1967; McArdle et al., 2002; Baltes et al., 2006).

The above characterization of cognitive abilities as defined across functional and temporal dimensions also informs research on cognition-mortality associations. For example, White and Cunningham (1988) proposed that Gc, which is relatively unaffected by aging, is more markedly affected by end-of-life processes (i.e., evincing sharper terminal decline and stronger associations with death) than Gf, which diminishes steadily with age and which presumably has “less room” to decline in the final years of life. However, recent work suggests that elevated mortality risk is more strongly associated with Gf than Gc (Ghisletta et al., 2006; Aichele et al., 2015, 2016) and that cognition-survival associations are quite robust across the adult lifespan (e.g., Bosworth et al., 1999; Thorvaldsson et al., 2006). However, if it is true that declines in crystallized abilities primarily manifest in close proximity to death, such declines may be especially prone to underestimation due to attrition (i.e., because individuals with sharp declines in Gc are more likely to drop out of a study due to illness and death). This suggests that understanding changes in different cognitive abilities during adulthood depends to some extent on how cognitive performance is modeled in relation to mortality risk.

#### Cognitive Performance of Survivors vs. Decedents

Most early studies of end-of-life changes in cognition assessed ability on one occasion, with follow-up recording of deaths, but no other events, at a single census point. Performance of survivors was then compared to that of decedents. Outcomes from these studies typically showed that decedents performed worse than survivors (e.g., Bosworth et al., 1999; Small et al., 2003). An important drawback of this methodology is that choice of census date influences group sample composition (i.e., at later census dates, there will be smaller percentages of survivors), and the sole dichotomous nature of survival status defined in this way ignores individual differences in length of survivorship (participants who died shortly before the census date are labeled similarly to those who died much earlier). This means that estimated cognition-mortality effects may either be attenuated or amplified contingent on choice of census date. Obtaining additional mortality-related information (e.g., changes in health status or illness-related dropout) has been shown to partially remedy these shortcomings (Rabbitt et al., 2005). However, an inherent limitation to this methodology is that it cannot accommodate information about individual differences in age at death or censoring due to survivorship.

#### Estimation of Cognitive Change in the Presence of Attrition

Longitudinal multilevel models (MLM) and structural equation models (SEM) are currently preferred by many researchers for estimating cognitive change. In studies of older adults, these models are usually applied to data with missing observations due to attrition (i.e., death or dropout). Not accounting for this missingness can contribute to underestimation of cognitive declines because higher functioning individuals remain in the study longer, giving an appearance of less decline and/or improved average functioning over time (a selection effect).

If attrition-related missingness is not directly contingent on differences in cognitive functioning (i.e., the outcome variable is not itself a source of attrition), then the data are considered missing at random (MAR), and, under maximum likelihood (ML) estimation, inclusion of covariates informative of death or dropout (e.g., smoking, general health status) can provide unbiased estimates of cognitive change. However, to the extent that missingness is directly contingent on differences in cognitive ability, then the data are considered to be missing not at random (MNAR), and unbiased estimates of longitudinal cognitive change can be obtained only if modeled conditionally on the corresponding basis for non-random attrition, e.g., death (Baraldi and Enders, 2012; Rouanet et al., 2017).

A related and highly debated issue in the application of longitudinal mixed models for estimating changes in health (and by extension cognitive ability) when attrition may be due to death is that such models assume an “immortal cohort” (Dufouil et al., 2004; Kurland et al., 2009; Wen and Seaman, 2018). That is, mixed models estimated under ML impute missing information following death (i.e., for decedents). Thus, it has been argued that the estimated average changes cannot be used for making valid inferences about the broader population (wherein all individuals remain alive) but rather only within the “immortal cohort” population assumed by the model. Attempts to remedy this problem initially took the form of weighting strategies and sensitivity analyses (Dufouil et al., 2004), but more recent innovation may now allow for estimating a conditional profile valid for “mortal” population inference, without separation of decedents’ data, by jointly modeling the longitudinal outcome and semi-competing event times of dropout and death (Li and Su, 2018). In either case, person-specific effects partialed from the group average trajectory (i.e., random or intraindividual effects in multilevel models) may still be validly interpreted at the population level and thus useful for inferential and predictive purposes (Rouanet et al., 2017).

#### Joint Longitudinal and Time-to-Event Models

Joint longitudinal-survival models typically combine a MLM with a proportional hazards survival model, which are estimated concurrently within a single statistical framework (Henderson et al., 2000; Tsiatis and Davidian, 2004; Rizopoulos et al., 2014; Li and Su, 2018). This means that missingness in longitudinal scores is accounted for by differences in mortality-risk (i.e., survival status and survival time), and missing time of death information for survivors is accounted for by differences in longitudinal performance. In other words, joint models are a useful approach when longitudinal information is MNAR conditional on the event of interest (e.g., death).^1^ Joint longitudinal-survival modeling has seen increasing use in biomedical research (e.g., Abdi et al., 2013; Thabut et al., 2013; Núñez et al., 2014; Li and Su, 2018), but it has very rarely been applied in research on adult developmental cognitive change (e.g., Ghisletta et al., 2006; Muniz-Terrera et al., 2011, 2018). Although the focus of these latter studies was to predict mortality risk contingent on differences and changes in cognitive abilities, it is informative to compare how longitudinal estimates of cognitive performance may differ when estimated in standalone MLM vs. when estimated in joint models using “real world” data, even if the group-level longitudinal parameter estimates (fixed effects) from these models arguably pertain to immortal cohorts.

Toward this end, we could identify only three prior studies that reported estimates of cognitive changes from both standalone longitudinal and joint longitudinal-survival modeling procedures. The first of these studies looked at Alzheimer’s disease (AD) onset as the target event in a sample of 398 Swedish older adults. Intercepts and slopes of memory performance differed very little across frameworks, with slightly larger standard errors when estimated with the joint models (McArdle et al., 2005). In the remaining studies (both looking at death as the target event, and both using data from the Swedish Twin Registry; *N* = 618 and *N* = 551, respectively), estimates for intercepts of cognitive performance differed little, and slope parameters were non-significant, across frameworks. There was a very slight reduction in the standard error of the intercept in joint analysis from the earlier of these studies (Muniz-Terrera et al., 2011, 2018). Taken together, these results indicate at most slight differences in magnitude and accuracy of longitudinal parameter estimates in standalone vs. joint models. However, with so few comparative studies from which to draw conclusions (i.e., three studies, all based on samples of Swedish participants), it remains worth asking (a) whether different results might be found in similar comparative studies of different populations and (b) whether the inclusion of health-related covariates may have mitigated differences across frameworks (i.e., as might be expected under MAR).

### Longitudinal Cognitive Performance as Predictive of Survival

Studies in cognitive epidemiology have shown that cognitive performance predicts differences in mortality risk in later adulthood (e.g., Anstey et al., 2001; Whalley and Deary, 2001; Bosworth and Siegler, 2002). Various interpretations have been given for cognition-mortality associations, and these are not necessarily mutually exclusive. For example, higher childhood IQ may influence later socioeconomic status and access to health care (and hence reduced mortality risk). Alternatively, associations between declines in cognitive function and differences in mortality risk may be mediated by an underlying health condition, such as cerebrovascular illness. In modeling cognition-mortality associations, it makes sense to place cognitive performance on the predictor side of the equation and survival status on the outcome side—if for no other reason than temporal precedence (death being final)—whether cognitive performance is viewed as a diagnostic indicator or as playing a causal role.

In previous work using data from the Manchester Longitudinal Study of Cognition (Rabbitt et al., 2004), we showed that lower baseline cognitive performance (intercepts) of Gc and Gf, and sharper decline (negative linear slope) in Gf, predicted increased mortality risk (Aichele et al., 2015). These results were based on a two-stage procedure in which longitudinal cognitive parameters (intercepts, slopes) were estimated prior to their inclusion as predictors in survival analysis. However, if Gc exhibits decline only in the terminal stage (White and Cunningham, 1988), a significant association between decline in Gc and mortality risk would more likely be found if longitudinal and survival processes were jointly estimated (i.e., to account for the effect of mortality-related dropout on estimated decline in Gc). This line of reasoning also applies to Gf to the extent that mortality-related declines in Gf do not overlap declines characterized by aging alone.

Testing these assumptions requires comparing two-stage vs. joint model estimates of cognition-mortality associations. We could identify only two prior longitudinal cognitive studies reporting results both from two-stage and joint longitudinal/time-to-event models. In the first of these studies (McArdle et al., 2005), joint modeling produced more accurate estimates (smaller standard errors) for the effects of cognitive ability on the target event (AD onset). Whereas slope-event estimates were non-significant across frameworks, there was sign flipping in the significant associations between intercepts of cognitive performance and disease onset across standalone (positive association) and joint (negative association) frameworks. In other words, the standalone model showed that better baseline memory predicted higher risk for AD onset, whereas the joint model showed that better baseline memory predicted lower risk for AD onset (a more theoretically admissible outcome). This suggests a selection effect such that AD-related dropout may have biased the estimate (and interpretation) of the memory-AD association in the standalone model (and hence a reason to prefer the joint framework).

The second study included eight cognitive measures (Ghisletta et al., 2006). In the two-stage analyses, none of the cognitive variables significantly predicted mortality risk. In the joint analyses, better baseline ability, and less decline in ability, predicted increased survival time across nearly all cognitive tasks, even when conditioned on age, sex, and socio-economic differences. Although these results highlight the joint modeling approach as potentially critical for accurate estimation of cognition-mortality associations, results from two-stage vs. joint models in this study cannot be directly compared because the former were based on models in which cognitive variables were analyzed simultaneously as predictors, whereas in the latter, the predictive effects of the cognitive variables were estimated independently.

Taken together, outcomes from these two studies indicate that estimated associations between cognitive trajectories and event incidence (whether AD onset or death) may be affected with respect to their accuracy (standard errors) and their magnitude and direction of effect. However, with scant extant research in this area, similar comparative studies are needed before firmer conclusions and recommendations for modeling strategies can be made.

### Potential Implications for Public Health

Prevalence rates for cognitive impairment in later life are 15–20%, with more severe forms (i.e., dementias) affecting an estimated 5–7%, of adults age 60 years and over. Cost of care currently approaches $1 trillion (USD) globally (World Health Organization, 2017). Thus, it is critically important to identify cost-effective, non-pharmacological strategies for addressing mental declines in later life. At present, it is thought that well-preserved knowledge and skills (i.e., crystallized abilities) can to some extent compensate for other cognitive declines, such as in memory and abstract reasoning, that are more strongly influenced by mortality-related pathologies (Stern, 2009). A clearer understanding of the extent to which these abilities are differentially sensitive to mortality risk may thus allow for more accurately gauging the potential of crystallized abilities to serve as compensatory factors.

### The Present Study

In a large-sample repeated-measures study of middle-aged and older adults, we compared two-stage vs. joint procedures for estimating longitudinal cognition-mortality associations. We estimated these models both with and without adjustment for survival-related covariates (smoking and self-rated health). We expected that differences across statistical frameworks in estimated average cognitive change and in cognition-mortality associations would be most evident in unadjusted models. We expected to observe sharper declines in cognitive abilities, stronger cognition-mortality associations, and improved estimation accuracy (narrower credible intervals) in results from joint analyses compared to results from two-stage analyses.

## Materials and Methods

Data for the analyses came from the Manchester Longitudinal Study of Cognition (MLSC). The broader aims and methods of the MLSC have previously been described at length (Rabbitt et al., 2004). We therefore briefly summarize participants and measures, whereas we describe statistical methods in greater detail. The MLSC was approved by the Department of Psychology Internal Ethics Committee, University of Manchester; by the University of Manchester Ethics Committee; and by the Greater Manchester National Institute for Health Trust Ethics Committee.

### Participants

Participants were recruited by magazine, radio, or television advertisements. A first wave of participants entered the MLSC in 1983, with subsequent cohorts recruited yearly until 1993. Cognitive testing was carried out until 2003. Data were selected from participants who completed one or more cognitive assessments of both Gc and Gf tasks, who were assessed between the ages of 50 and 87 years, who had complete information for mortality status (i.e., dead vs. alive; and if dead, age at death) as of August, 2012, and who were free from dementia at time of assessment.

In the original sample, some individuals were observed prior to age 50 years and/or after age 87 years. Here, we set a minimum cutoff of 20 observations (*n* = 20) per time point to safeguard against leveraging effects from outliers, resulting in the current age range. Individuals with severe visual or auditory handicaps were excluded from the study. There were 4,197 women (70.5%) and 1,757 men (29.5%), giving a combined *N* = 5,954. Median age at study entry was 65.0 years [interquartile range (IQR) = 60.0–70.0 years]. In general, participants had higher income and education levels (38% completed one or more years of college) than U.K. averages, but there were substantial numbers of participants with lower incomes and less education than the U.K. population average (Rabbitt et al., 1993). Of participants, 1470 (24.7%) were tobacco smokers. Self-rated health (SRH) scores (scaled 1 = poor to 5 = excellent) showed that participants generally considered themselves to be healthy at study entry (Median = 4, IQR = 3–5).

Mortality information (dates and proximate causes for all deaths between 1983, when the study began, and August 2012, the most recent update) was obtained from a search of death certificates performed by Her Majesty’s General Registry Office. Of participants selected for the current analyses, there were 4,453(74.8%) deceased and 1,501 (25.2%) survivors as of 2012. For deceased, median age at study entry was 66.0 years (IQR = 62.0–71.0 years). Median age at death was 84.0 years (IQR = 77.0–89.0 years). Median time-to-death from study entry was 17.0 years (IQR = 10.0–22.0 years), and median time-to-death from last completed cognitive assessment was 10.0 years (IQR = 5.0–16.0 years).

### Cognitive Measures

Cognitive data for the current analyses were obtained from measures of crystallized intelligence (Gc) and fluid intelligence (Gf). For each of these domains, three separate tasks were administered shortly after induction into the study and subsequently up to three times, on average at 4-year intervals (IQR = 3.0–5.0-year intervals). Thus, each individual completed up to 4 cognitive testing occasions over a period of approximately 12 years. Cognitive measures were selected on the basis that they were appropriate for assessment of cognitive change in samples of older adults according to lifespan developmental theory (Baltes et al., 2006), are well-known and documented in the empirical literature, have good psychometric properties, and could be administered by pencil-and-paper for effective assessment within a large sample.

Gc was measured by the Raven (1965) Mill Hill Vocabulary A and B (synonyms and word definitions) subtests and by the Wechsler Adult Intelligence Scale-Revised (WAIS-R) vocabulary scale (Wechsler, 1986). Gf was assessed by the Heim (1970) AH4-1 and AH4-2 tasks (logic, arithmetic, number series, verbal and visuospatial objects comparisons) and the Cattell and Cattell (1960) Culture Fair Intelligence Test (2A or 2B, at alternating occasions). Raw scores for each measure were rescaled to be centered at the first testing occasion, with mean = 50 and *SD* = 10 (T metric). We used longitudinal structural factor analyses with strict factorial invariance to aggregate data from individual tasks as factor scores (i.e., Gc, Gf) across measurement occasions, as described in Aichele et al. (2015). Factor scores were re-centered during estimation with mean = 0 at the first occasion. On average, participants completed 2 cognitive assessments (*M* = 2.2). Median time in study (across cognitive testing) was 4.0 years (IQR = 0.0–8.0 years). Longitudinal summary statistics based on measurement occasion (observations, age, and cognitive performance) are shown in Table 1.

**TABLE 1:** Summary information by cognitive measurement occasion.

	Cognitive measurement occasion			
	T1	T2	T3	T4
**Women**				
*n*	4,197	2,675	1,554	792
Deaths (cumulative)	0	170	334	434
Age (median)	65.0	69.0	72.0	75.0
Gc (mean)	−0.06	0.20	0.14	0.40
Gf (mean)	−0.08	0.19	0.25	0.34
**Men**				
*n*	1,757	1,109	603	295
Deaths (cumulative)	0	156	292	374
Age (median)	67.0	70.0	74.0	76.0
Gc (mean)	0.17	0.42	0.34	0.43
Gf (mean)	0.17	0.42	0.34	0.52

*Age = chronological age in years at time of cognitive assessment. Age ranges at each measurement occasion are not shown as these were capped between 50 and 87 years for the current analyses. Number of deaths were approximated based on age at death (if known) relative to last observed age at assessment. If age at death was = 4 years from last assessment age, then absence at follow-up was attributed to death rather than dropout. Gc, crystallized intelligence, standardized factor score; Gf, fluid intelligence, standardized factor score. Cognitive factor scores were scaled based on the mean and standard deviation (based on combined scores of women and men) at first measurement occasion. Standard deviations for cognitive scores were near 1.0 across occasions and so are not reported.*

### Statistical Analyses

#### Trajectories of Cognitive Change

In a first series of analyses, we estimated cognitive changes using multilevel models (MLM; Laird and Ware, 1982). Three MLM were specified, as described by the following latent change parameters: (a) intercept-only model (intercepts as fixed and random effects), (b) linear change model (intercept and linear change component as both fixed and random effects), and (c) quadratic change model (linear change model plus quadratic change component as a fixed effect only because few participants completed more than three assessments). Consistent with previous comparative studies noted in the introduction, we modeled cognitive change based on chronological age in years (centered at age 65 years, which was the median age at testing; for a similar approach, see Gerstorf et al., 2013).

We applied the MLM to data from all participants (i.e., inclusive of both survivors and decedents) and then to data only from decedents (to examine the potential selection effect of excluding survivors). Analyses were conducted separately by biological sex (men, women) because we previously demonstrated non-proportional mortality hazards for women and men (Aichele et al., 2015), and we wanted the standalone MLM to be consistent with the joint longitudinal-survival models. MLM models were first estimated using R statistical software (version 3.3.2; R Core Team, 2016) with package nlme (Pinheiro et al., 2016) with maximum likelihood estimation. We compared model fit using standard chi-square tests of changes in log-likelihood. Note that models selected based on these comparisons were used simply for providing starting values required by the joint modeling software, JMBayes, described below (Rizopoulos, 2012, 2016). JMBayes uses Monte Carlo (MCMC) estimation with Gibbs sampling. Therefore, for comparative purposes, we re-analyzed the best-fitting MLM both in standalone and joint longitudinal-survival analytical frameworks using JMbayes MCMC estimation with non-informative priors.

Equation (1) shows the specification for the quadratic change model, under which intercept-only and linear change models were nested:

(1) $$\begin{array}{l} Y_{ti}=B_{0}+B_{1}\cdot Age_{ti}+B_{2}\cdot Age_{}^{2}{}_{ti}^{}\\ \;+(u_{0\text{i}}+u_{1\text{i}}\cdot Age_{ti}+e_{ti}) \end{array}$$

In this equation, *Y*_ti_ is the cognitive factor score (Gc, Gf) at age *t* for individual *i*; *B*_0_, *B*_1_, and *B*_2_ correspond to fixed effects of intercept, linear change and quadratic change in chronological age in years; *u*_0i_ and *u*_1i_ are random effects for intercept and linear change, respectively, and *e*_ti_ corresponds to residual component (random effects for quadratic change could not be estimated under ML, and we did not include them in models estimated with MCMC). To the best fitting models, we then added covariates (as fixed effects) that were later included in the survival models (see below). These were age at study entry (also centered at 65 years), the interaction of age at study entry with linear change in age, smoking (yes/no), and SRH at study entry.^2^

(2) $$\begin{array}{l} Y_{ti}=B_{0}+B_{1}\cdot Age_{ti}+B_{2}\cdot Age_{}^{2}{}_{ti}^{}+B_{3}\cdot StartAge_{i}\\ \;+B_{4}\cdot StartAge_{i}\cdot Age_{ti}+B_{5}\cdot Smoking_{i}+B_{6}\cdot SRH_{i}\\ \;+(u_{0\text{i}}+u_{1\text{i}}\cdot Age_{ti}+e_{ti}) \end{array}$$

Equation (2) thus adds to Equation (1): *B*_3_, the effect of age at study inception (*StartAge*_i_); *B*_4_, the interaction between starting age and age-related linear change; and B_5_ and B_6_, the effects of being a smoker and of SRH, respectively.

#### Cognition-Survival Associations

We estimated cognition-survival associations by two approaches. First, we used a two-stage method in which we estimated individuals’ scores for intercepts and linear changes for each cognitive ability from the best-fitting MLM models (i.e., MCMC-estimated individual random effects), and we then used those scores to predict mortality risk in a Cox proportional hazards (PH) modeling framework (Cox, 1972). The Cox PH model specifies a multiplicative association between covariates and the target event (in this case death), formulated as follows:

(3) $$h_{\text{i}}\,\left(t\right)=h_{0}\,\left(t\right)\,e\,x\,p\,\left(\gamma \,w_{\text{i}}\right),$$

where *h*_i_(*t*) is the cumulative hazard up to time *t* for individual *i*, *h*_0_(*t*) is the baseline hazard function at time *t*, and *w*_i_ is a vector of baseline predictors with corresponding regression coefficients γ (described below). Survival models in the two-stage procedure were estimated with a MCMC approach using R package spBayesSurv (Zhou et al., 2017) to ensure that both 2-stage and joint modeling were estimated with MCMC. MCMC estimation was run with 20,000 iterations per model with a burn-in phase of 3000 iterations. Covariates included in the survival models are described in the following section.

#### Joint Longitudinal-Survival Models

We next conducted a series of joint longitudinal-survival analyses. Compared to a two-stage approach, joint modeling reduces bias in longitudinal parameters by concurrently accounting for sources of informative dropout (e.g., missingness related to mortality risk) during estimation. It does this by linking the longitudinal and survival statistical frameworks through shared parameterization (Henderson et al., 2000). The defining feature of the joint model is that longitudinal and survival data are modeled simultaneously with respect to a conditional joint density, rather than separately with two independent marginal densities (Rizopoulos et al., 2014). For fitting the joint models, we used the R package JMbayes (Rizopoulos, 2012, 2016). JMbayes fits joint models under a Bayesian approach using MCMC estimation (with starting values obtained from standalone MLM and CoxPH models). We again specified MCMC estimation to run with 20,000 iterations per model and with a burn-in phase of 3000 iterations). We evaluated model convergence in reference to common MCMC diagnostic plots (trace, auto-correlation, and kernel density).

We fit joint models using the JMbayes “shared random effects” association structure, which links the random effects (individual deviations from the trajectory parameters specified in the longitudinal sub-model) to the survival process. For this study, random effects were limited to individual intercept and linear change terms (specified in the longitudinal model of Equation 1), giving the following equation for the initial JMbayes survival sub-model:

(4) $$h_{\text{i}}\,\left(t\right)=h_{0}\,\left(t\right)\,e\,x\,p\,\left(\gamma \,w_{\text{i}}+\alpha_{1}\,u_{0\,i}+\alpha_{2}\,u_{1\,i}\right),$$

which expands on Equation (2) by adding α_1_ and α_2_, the predictive effects corresponding respectively to the random intercept, u_0i_, and random linear change component, u_1i_, of the individual cognitive trajectory. Thus, α_1_ and α_2_ are the shared parameters for the joint longitudinal-survival models (Rizopoulos, 2016). For interested readers, we provide as Supplementary Materials working code (S1) and simulated data (S2) to demonstrate how to implement JMbayes. A longer tutorial is also available (Cekic et al., in press). Further discussion regarding implementation of two-stage vs. joint approaches can be found in Guo and Carlin (2004) and Rizopoulos (2012).

For both two-stage and joint longitudinal-survival analyses, we first estimated mortality risk only as a function of the cognitive predictors (intercepts and linear changes for Gc and Gf). These were the shared parameters for the joint longitudinal-survival models, and we wanted to look at their predictive influence in isolation. We then expanded the analyses by further including covariates that from previous work (Aichele et al., 2015, 2016) we knew to be comparatively strongly predictive of mortality risk (age at study entry, smoking status, and SRH, thereby expanding Equation 3). Note that we did not include covariates related to socioeconomic status (i.e., education, occupational class) as in prior work we found these variables to have a minor influence on mortality risk in this sample. Survival analyses were run independently by sex because we previously showed non-proportional hazards across men and women. In total, there were 16 survival analyses using data from all participants, with permutations defined as: Statistical framework (2-stage/Joint) ^∗^ sex(F/M) ^∗^ cognitive domain (Gc/Gf) ^∗^ covariates (no/yes).

## Results

The MLM estimated with ML all converged. Diagnostic plots for models re-estimated with MCMC indicated that 20,000 iterations were largely sufficient for obtaining stable parameter estimates.

### Trajectories of Cognitive Change

**FIGURE 1:**
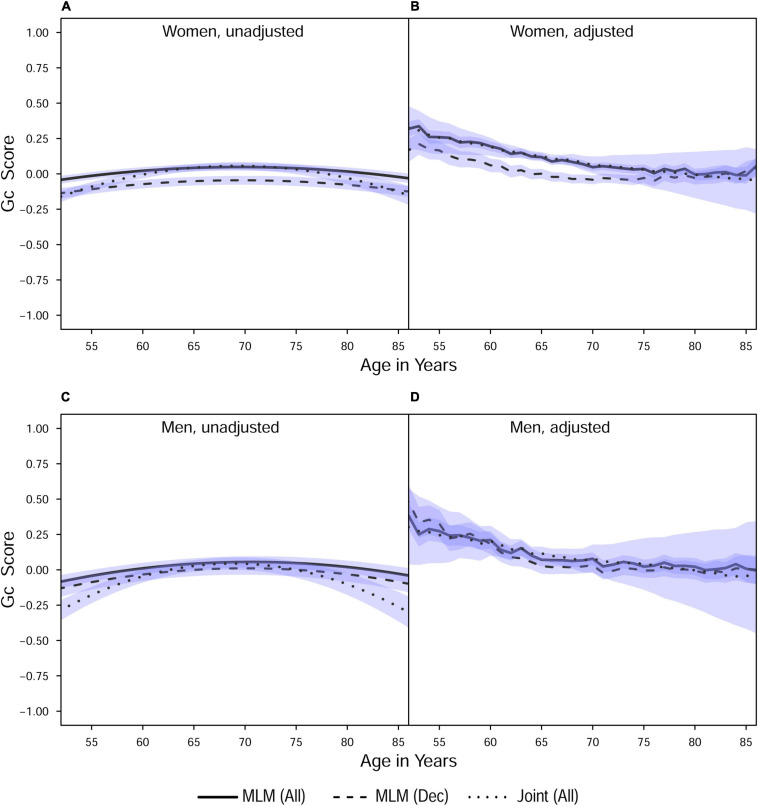
Estimated average changes in crystallized intelligence (Z-scaled) by chronological age. **(A,C)** Trajectories unadjusted for starting age and health-related covariates. **(B,D)** Trajectories adjusted for differences in starting age, SRH, and tobacco smoking. MLM, standalone, multilevel model trajectories; Joint, joint longitudinal-survival model trajectories; All, data from decedents and survivors; Dec, data only from decedents. Shaded regions indicate 95% credible intervals for the group average trajectories.

**FIGURE 2:**
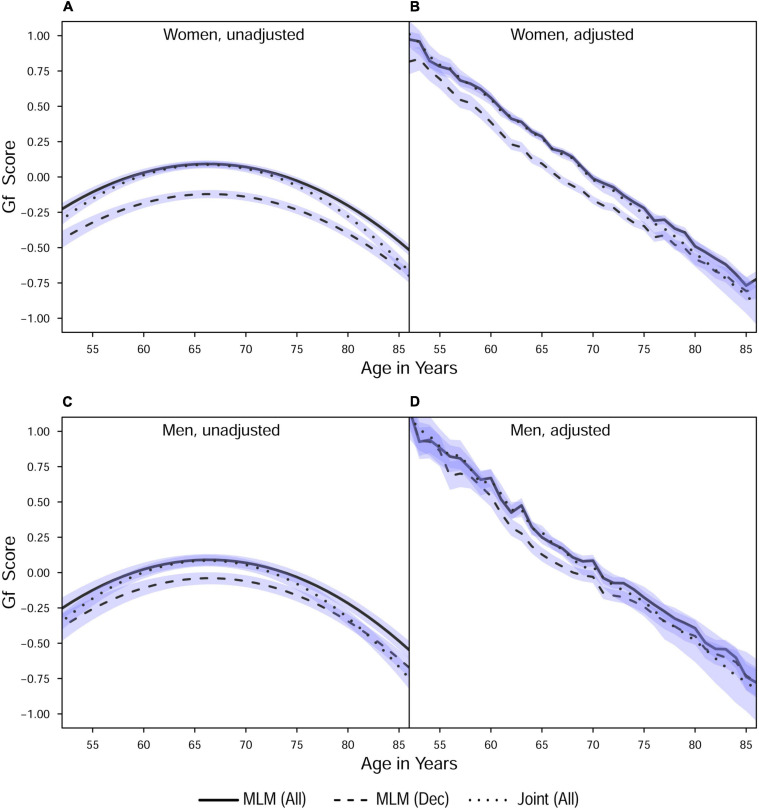
Estimated average changes in fluid intelligence (Z-scaled) by chronological age. **(A,C)** Trajectories unadjusted for starting age and health-related covariates. **(B,D)** Trajectories adjusted for differences in starting age, SRH, and tobacco smoking. MLM, standalone, multilevel model trajectories; Joint, joint longitudinal-survival model trajectories; All, data from decedents and survivors; Dec, data only from decedents. Shaded regions indicate 95% credible intervals for the group average trajectories.

Initial model fit comparisons based on changes in log-likelihood are reported as Supplementary Materials (S3). Fit statistics for standalone MLM with quadratic changes (the best-fitting models) estimated using MCMC are provided in Table 2. Explained variation for multilevel models is given as *pseudo-R*^2^, which was estimated as the proportional reduction in residual variance relative to that from an unconditional means model (i.e., fixed and random intercepts only), consistent with the method proposed by Snijders and Bosker (1999). Estimated trajectories of cognitive change are shown in Figure 1 (Gc) and Figure 2 (Gf). Estimates for fixed and random effects from the standalone MLM and from the longitudinal components of the joint analyses are provided in Tables 3–6. To facilitate comparison of estimated trajectories across analytic frameworks, we also report linear changes in cognition approximated as changes in standardized scores per decade for models with covariates (ΔZ/10y; Table 5, last column).

**TABLE 2:** Fit statistics from multilevel models with quadratic change.

Sample	N	Obs	Model	Deviance	BIC	*Pseudo-R* ^2^
**Crystallized intelligence**						
Women, all	4,197	9,202	QC	48,063	48,127	0.12
	3,801	8,658	QC.c	44,138	44,238	0.16
Women, decedents	2,978	6,090	QC	32,597	32658	0.13
	2,681	5,694	QC.c	29,756	29,852	0.19
Men, all	1,757	3,758	QC	19,748	19,806	0.17
	1,615	3,559	QC.c	18,289	18,379	0.23
Men, decedents	1,475	3,039	QC	16,248	16,304	0.18
	1,352	2,875	QC.c	14,982	15,069	0.26
**Fluid intelligence**						
Women, all	4,191	9,196	QC	49,367	49,430	0.57
	3,799	8,656	QC.c	44,188	44,288	0.65
Women, decedents	2,973	6,085	QC	33,213	33,274	0.58
	2,679	5,692	QC.c	29,757	29,852	0.66
Men, all	1,754	3,755	QC	20,684	20,742	0.61
	1,613	3,557	QC.c	18,784	18,874	0.69
Men, decedents	1,473	3,037	QC	16,904	16,960	0.63
	1,351	2,874	QC.c	15,402	15,490	0.71

*QC, quadratic change model; QC.c, quadratic change with covariates for age at study entry, starting age ^∗^ linear change interaction, smoking status, and self-rated health; Obs, observations; BIC, Bayesian information criterion. Pseudo-R^2^ was estimated as proportional reduction in residual variance from an unconditional means model (Snijders and Bosker, 1999).*

**TABLE 3:** Fixed effects from quadratic change models (unadjusted).

		Fixed effects								
Analysis	Data	Intercept			Linear change			Quadratic change		
**Crystallized intelligence (women)**										
MLM	All	−0.19	[−0.45,	0.07]	0.02	[0.02,	0.03]	0.00	[0.00,	0.00]
MLM	Dec	−1.04	[−1.35,	−0.71]	0.02	[0.01,	0.04]	0.00	[0.00,	0.00]
Joint	All	−0.18	[−0.46,	0.08]	0.06	[0.04,	0.08]	−0.01	[−0.01,	−0.01]
**Crystallized intelligence (men)**										
MLM	All	1.96	[1.57,	2.37]	0.04	[0.02,	0.06]	0.00	[0.00,	0.00]
MLM	Dec	1.58	[1.13,	2.02]	0.04	[0.02,	0.06]	0.00	[−0.01,	0.00]
Joint	All	1.82	[1.41,	2.26]	0.08	[0.04,	0.12]	−0.01	[−0.01,	−0.01]
**Fluid intelligence (women)**										
MLM	All	0.12	[−0.13,	0.38]	0.03	[0.02,	0.05]	−0.01	[−0.01,	−0.01]
MLM	Dec	−1.71	[−2.01,	−1.42]	0.04	[0.02,	0.06]	−0.01	[−0.01,	−0.01]
Joint	All	0.08	[−0.18,	0.34]	0.04	[0.03,	0.05]	−0.02	[−0.02,	−0.02]
**Fluid intelligence (men)**										
MLM	All	2.71	[2.28,	3.14]	0.04	[0.01,	0.07]	−0.02	[−0.02,	−0.01]
MLM	Dec	1.56	[1.11,	2.01]	0.04	[0.01,	0.08]	−0.02	[−0.02,	−0.01]
Joint	All	2.66	[2.24,	3.08]	0.05	[0.03,	0.07]	−0.02	[−0.02,	−0.02]

*MLM, multilevel model with age as time metric; Joint, joint longitudinal-survival model using age as time metric. For all models, time was scaled in years. All, using data from all participants; Dec, using data from decedents. Brackets denote 95% CI from MCMC estimation.*

**TABLE 4:** Random effects from quadratic change models (unadjusted).

		Random effects											
Analysis	Data	Intercept			Linear change			*r* (I,LC)			Residual		
**Crystallized intelligence (women)**													
MLM	All	80.50	[77.17,	84.41]	0.01	[0.00,	0.01]	0.62	[0.52,	0.73]	1.08	[1.04,	1.13]
MLM	Dec	79.24	[75.26,	83.54]	0.01	[0.01,	0.01]	0.53	[0.43,	0.64]	1.05	[0.99,	1.11]
Joint	All	74.27	[70.75,	77.99]	0.32	[0.30,	0.34]	0.07	[0.03,	0.11]	1.12	[1.07,	1.17]
**Crystallized intelligence (men)**													
MLM	All	75.91	[70.62,	81.07]	0.01	[0.01,	0.01]	0.62	[0.48,	0.77]	1.06	[0.99,	1.14]
MLM	Dec	77.51	[71.86,	84.12]	0.01	[0.01,	0.01]	0.61	[0.47,	0.75]	1.06	[0.98,	1.15]
Joint	All	69.50	[64.29,	74.80]	0.47	[0.44,	0.51]	0.08	[0.02,	0.14]	1.11	[1.01,	1.20]
**Fluid intelligence (women)**													
MLM	All	71.97	[68.80,	75.02]	0.05	[0.04,	0.05]	−0.07	[−0.13,	−0.01]	1.06	[1.01,	1.12]
MLM	Dec	65.10	[61.51,	68.88]	0.05	[0.05,	0.06]	−0.12	[−0.19,	−0.05]	1.12	[1.05,	1.20]
Joint	All	71.75	[68.56,	75.02]	0.09	[0.08,	0.09]	−0.07	[−0.12,	−0.03]	0.95	[0.90,	1.00]
**Fluid intelligence (men)**													
MLM	All	75.04	[70.18,	80.69]	0.06	[0.05,	0.07]	−0.05	[−0.14,	0.04]	1.18	[1.08,	1.29]
MLM	Dec	69.69	[64.09,	75.46]	0.07	[0.06,	0.08]	−0.08	[−0.18,	0.02]	1.24	[1.13,	1.38]
Joint	All	74.09	[69.07,	79.43]	0.12	[0.11,	0.14]	−0.06	[−0.13,	0.01]	1.04	[0.96,	1.13]

*MLM, multilevel model with age as time metric; Joint, joint longitudinal-survival model using age as time metric. For all models, time was scaled in years. All, using data from all participants; Dec, using data from decedents; r(I,LC), correlation between intercept and linear change. Brackets denote 95% CI.*

**TABLE 5:** Fixed effects from quadratic change models (adjusted for starting age, self-rated health, and smoking).

Analysis	Data	Intercept			Linear change			Quadratic change			Start age			Start age × LC			Smoke^a^	SRH^a^	ΔZ/10y
**Crystallized intelligence (women)**																			
MLM	All	−6.68	[−7.91,	−5.38]	0.14	[0.12,	0.15]	−0.01	[−0.01,	−0.01]	−0.27	[−0.31,	−0.23]	0.02	[0.02,	0.02]	−1.55	1.67	−0.104
MLM	Dec	−7.20	[−8.82,	−5.49]	0.15	[0.12,	0.16]	−0.01	[−0.02,	−0.01]	−0.26	[−0.32,	−0.21]	0.02	[0.02,	0.03]	−1.57	1.64	−0.066
Joint	All	−0.27	[−0.56,	0.03]	0.16	[0.05,	0.27]	−0.01	[−0.01,	−0.01]	−0.28	[−0.32,	−0.24]	0.02	[0.01,	0.02]	*0.00*	*0.00*	−0.108
**Crystallized intelligence (men)**																			
MLM	All	−3.64	[−5.54,	−1.70]	0.17	[0.15,	0.19]	−0.01	[−0.02,	−0.01]	−0.29	[−0.35,	−0.23]	0.02	[0.02,	0.03]	−*0.27*	1.40	−0.101
MLM	Dec	−3.19	[−5.54,	−0.99]	0.19	[0.16,	0.22]	−0.02	[−0.02,	−0.02]	−0.34	[−0.42,	−0.27]	0.03	[0.02,	0.03]	−*0.45*	1.24	−0.126
Joint	All	1.93	[1.44,	2.42]	0.19	[0.00,	0.37]	−0.01	[−0.02,	−0.01]	−0.30	[−0.36,	−0.24]	0.02	[0.01,	0.02]	*0.02*	*0.00*	−0.104
**Fluid intelligence (women)**																			
MLM	All	−7.00	[−8.31,	−5.74]	0.23	[0.21,	0.25]	−0.03	[−0.03,	−0.03]	−0.77	[−0.81,	−0.73]	0.04	[0.04,	0.04]	−2.25	1.74	−0.525
MLM	Dec	−7.03	[−8.53,	−5.65]	0.23	[0.21,	0.25]	−0.03	[−0.03,	−0.03]	−0.78	[−0.83,	−0.72]	0.04	[0.04,	0.05]	−2.22	1.59	−0.508
Joint	All	−0.50	[−0.75,	−0.26]	0.23	[0.16,	0.29]	−0.03	[−0.03,	−0.03]	−0.76	[−0.80,	−0.73]	0.04	[0.04,	0.04]	−*0.01*	*0.00*	−0.554
**Fluid intelligence (men)**																			
MLM	All	−3.90	[−5.81,	−2.07]	0.24	[0.21,	0.27]	−0.03	[−0.03,	−0.03]	−0.79	[−0.86,	−0.73]	0.04	[0.04,	0.05]	−1.57	1.78	−0.539
MLM	Dec	−3.89	[−5.93,	−1.84]	0.24	[0.21,	0.28]	−0.03	[−0.04,	−0.03]	−0.81	[−0.88,	−0.72]	0.05	[0.04,	0.06]	−1.06	1.62	−0.537
Joint	All	2.70	[2.26,	3.12]	0.23	[0.13,	0.33]	−0.03	[−0.03,	−0.03]	−0.78	[−0.84,	−0.72]	0.04	[0.04,	0.05]	*0.02*	*0.00*	−0.567

*MLM, multilevel model with age as time metric; Joint, joint longitudinal-survival model using age as time metric. For all models, time was scaled in years. All, using data from all participants; Dec, using data from decedents; Start Age, participant age at start of study, offset from 65; SRH, self-rated health; ΔZ/10y, linear change per decade in the respective standardized cognitive scores. Brackets indicate 95% CI. ^a^CI for Smoker and SRH are not shown due to space considerations; The italicized values indicate credible intervals overlapping 0.*

**TABLE 6:** Random effects from quadratic change models (adjusted for starting age, self-rated health, and smoking).

Analysis	Data	Intercept			Linear change			*r* (I,LC)			Residual		
**Crystallized intelligence (women)**													
MLM	All	73.60	[70.40,	76.97]	0.01	[0.00,	0.01]	0.67	[0.59,	0.77]	1.03	[0.99,	1.08]
MLM	Dec	73.94	[70.08,	77.99]	0.01	[0.01,	0.01]	0.56	[0.46,	0.67]	0.98	[0.93,	1.04]
Joint	All	68.35	[65.31,	71.49]	0.32	[0.30,	0.34]	0.07	[0.03,	0.10]	1.10	[1.04,	1.15]
**Crystallized intelligence (men)**													
MLM	All	71.40	[66.44,	76.51]	0.01	[0.01,	0.01]	0.63	[0.49,	0.76]	0.98	[0.91,	1.05]
MLM	Dec	72.52	[67.07,	78.60]	0.01	[0.01,	0.01]	0.62	[0.50,	0.76]	0.95	[0.87,	1.03]
Joint	All	64.31	[59.67,	69.40]	0.44	[0.40,	0.48]	0.06	[0.01,	0.12]	1.05	[0.97,	1.14]
**Fluid intelligence (women)**													
MLM	All	53.55	[51.14,	56.16]	0.05	[0.04,	0.05]	0.04	[−0.01,	0.09]	0.86	[0.82,	0.91]
MLM	Dec	52.13	[49.21,	55.36]	0.06	[0.05,	0.06]	–0.03	[−0.10,	0.04]	0.90	[0.84,	0.97]
Joint	All	52.78	[50.44,	55.33]	0.08	[0.07,	0.09]	0.04	[−0.01,	0.08]	0.80	[0.76,	0.84]
**Fluid intelligence (men)**													
MLM	All	59.70	[55.51,	64.34]	0.06	[0.05,	0.07]	0.07	[−0.01,	0.15]	0.93	[0.86,	1.02]
MLM	Dec	59.05	[54.64,	63.95]	0.07	[0.06,	0.08]	0.04	[−0.05,	0.12]	0.97	[0.88,	1.08]
Joint	All	58.26	[54.17,	62.58]	0.12	[0.11,	0.13]	0.05	[−0.02,	0.12]	0.85	[0.79,	0.92]

*MLM, multilevel model with age as time metric; Joint, joint longitudinal-survival model using age as time metric. For all models, time was scaled in years. All, using data from all participants; Dec, using data from decedents; r(I,LC), correlation between intercept and linear change. Brackets denote 95% CI.*

In general, explained variation was higher for Gf than for Gc. Gf declined at a faster rate than Gc (the latter showed only slight declines in models adjusted for covariates). Men had higher levels of initial cognitive performance but subsequently showed steeper declines than women. Adjustment for covariates (age at study entry, smoking, and SRH) effectively removed downward curvature (i.e., accelerated declines in later life) that were visibly present in the unadjusted models. We previously reported similar outcomes from analyses of these data (Aichele et al., 2015), so we now turn our attention to the comparative analyses that are the basis for the current study.

#### Joint Longitudinal-Survival Models vs. Standalone MLM

To compare overall predictive accuracy across standalone vs. joint models, we first examined differences in residual (unexplained) variance (Tables 4, 6) rather than calculating *pseudo-R*^2^ values because the latter are not applicable for the joint models. Note that for the joint models, residual variance here pertains only to the longitudinal sub-model (not the survival sub-model). Specifically, we examined whether the point estimate for residual variance from the joint analysis fell within the 95% CI for residual variance from the corresponding MLM analysis. For Gc, residual variances were slightly higher in joint analyses, but they mostly fell within the 95% CI from standalone MLM. In contrast, for Gf, residual variances were lower in joint models and fell outside the 95% CI from standalone MLM. Thus, for Gf, joint models appeared to confer slightly improved overall estimation accuracy for the longitudinal parameters.

With respect to the average trajectories (Figures 1, 2), in unadjusted models, cognitive changes estimated in joint analyses showed comparatively more downward curvature (significantly steeper quadratic declines) than those from corresponding standalone models. These differences were more pronounced at ends of the age range where observations were sparse (and CI were correspondingly wider due to increased uncertainty). The trajectories estimated in standalone MLM vs. joint models only differed significantly at far ends of the age range in models unadjusted for differences in starting age, smoking, and SRH.

Comparing the fixed longitudinal parameter estimates in models adjusted for covariates (Table 5), estimates for intercepts and for the effects of smoking and SRH on Gc and Gf scores differed dramatically across standalone vs. joint frameworks. However, the average trajectories for Gc and Gf differed litter across frameworks—and in fact less so after adjustment for these covariates (Figures 1, 2). This indicates that explained variance in Gc and Gf scores was partitioned differently across the two frameworks: I.e., estimated effects of health-related predictors were offset by changes in the estimated intercepts in standalone models; whereas in joint models, conditioning on survival status negated any effects of health covariates, and intercepts remained unaffected. With respect to random effects, within-person variation in intercepts of Gc and Gf was reduced, and within-person variation in linear changes in Gc and Gf was amplified, in joint vs. standalone models, whether unadjusted (Table 4) or adjusted (Table 6).

#### Sample Selection: All Participants vs. Decedents

*Pseudo-R*^2^ values were 1%–3% higher for analyses restricted to data from decedents vs. those using data from all participants (Table 2). Trajectories for decedents indicated worse overall cognitive performance (lower intercepts) compared to trajectories estimated with data from all participants, but the 95% credible intervals (CI) for the average trajectories of each sample largely overlapped (i.e., differences were non-significant), especially at far ends of the observed age range—with the exception of the unadjusted Gc trajectories for women (Figure 1A) and the unadjusted Gf trajectories for women and men (Figures 2A, C). In other words, adjustment for differences in starting age and health-related covariates reduced differences in average cognitive declines across decedents vs. all participants.

### Cognition-Survival Associations

Mortality risk estimates are summarized in Table 7. Significant proportional hazards for Gc predictors emerged only in joint analyses. Better baseline Gc (higher intercepts) predicted reduced mortality risk in unadjusted and adjusted joint models for women but only in adjusted joint models for men. Estimated proportional hazards for Gc intercepts were more negative (stronger effect) and had narrower 95% CI (greater accuracy) when estimated in joint vs. two-stage analyses. Linear changes in Gc significantly negatively predicted mortality risk (i.e., less Gc decline was associated with lower mortality risk) only in unadjusted joint models. Raw estimates for the proportional hazards of linear changes in Gc from the corresponding two-stage models were stronger (more negative) but less accurate (wider 95% CI which overlapped null values). For Gf, better baseline ability (more positive intercepts) and less decline (less negative linear changes) predicted reduced mortality risk in both two-stage and joint analyses, with and without adjustment for health-related covariates. For Gf intercepts, magnitudes and 95% CI of the estimated proportional hazards differed little across two-stage and joint analyses. In contrast, proportional hazards for Gf linear changes were stronger (more negative) and more accurate (narrower 95% CI) when estimated in joint vs. two-stage analyses.

**TABLE 7:** Cognition-survival associations (all participants).

	Proportional hazards											HR (scaled)				
		
Model	re.I			re.LC			Start age			Smoker^a^	SRH^a^	re.I	re.LC	Start age	Smoker	SRH
**Crystallized intelligence in women (N = 4197, Deaths = 2978)**																
2stage	–0.008	[−0.021,	0.004]	–0.466	[−2.590,	1.749]						0.931	0.977			
2st.cov	0.001	[−0.014,	0.015]	–1.632	[−3.804,	0.521]	−0.037	[−0.043,	−0.031]	0.484	−0.243	1.005	0.914	0.769	1.622	0.824
Joint	–0.009	[−0.014,	−0.005]	–0.234	[−0.410,	−0.042]						0.924	0.877			
Jnt.cov	–0.013	[−0.017,	−0.007]	0.049	[−0.172,	0.278]	−0.044	[−0.051,	−0.038]	0.499	−0.248	0.901	1.028	0.745	1.647	0.825
**Crystallized intelligence men (N = 1757, Deaths = 1475)**																
2stage	0.004	[−0.014,	0.021]	–1.932	[−4.342,	0.590]						1.036	0.890			
2st.cov	0.010	[−0.008,	0.027]	–2.292	[−4.389,	0.122]	−0.037	[−0.046,	−0.029]	0.197	−0.250	1.083	0.861	0.772	1.218	0.813
Joint	0.000	[−0.006,	0.008]	–0.755	[−1.003,	−0.460]						0.999	0.596			
Jnt.cov	–0.009	[−0.017,	−0.001]	0.082	[−0.268,	0.390]	−0.042	[−0.051,	−0.032]	0.188	−0.265	0.930	1.056	0.763	1.207	0.806
**Fluid intelligence women (N = 4191, Deaths = 2973)**																
2stage	–0.007	[−0.011,	−0.002]	–0.980	[−1.262,	−0.702]						0.946	0.886			
2st.cov	–0.013	[−0.018,	−0.007]	–0.909	[−1.184,	−0.634]	−0.038	[−0.044,	−0.031]	0.473	−0.245	0.914	0.885	0.765	1.604	0.822
Joint	–0.005	[−0.010,	−0.001]	–1.294	[−1.526,	−1.058]						0.960	0.683			
Jnt.cov	–0.012	[−0.018,	−0.007]	–1.036	[−1.304,	−0.790]	−0.046	[−0.053,	−0.040]	0.495	−0.265	0.916	0.745	0.735	1.641	0.814
**Fluid intelligence men (N = 1754, Deaths = 1473)**																
2stage	–0.009	[−0.016,	−0.003]	–0.854	[−1.202,	−0.502]						0.924	0.885			
2st.cov	–0.015	[−0.022,	−0.008]	–0.756	[−1.104,	−0.419]	−0.038	[−0.047,	−0.030]	0.181	−0.253	0.894	0.889	0.768	1.198	0.810
Joint	–0.010	[−0.017,	−0.002]	–1.266	[−1.561,	−0.974]						0.922	0.642			
Jnt.cov	–0.016	[−0.024,	−0.009]	–0.986	[−1.249,	−0.684]	−0.045	[−0.056,	−0.036]	0.185	−0.278	0.883	0.714	0.745	1.203	0.797

*2stage = two-stage survival analysis with only cognitive predictors. Joint = joint longitudinal-survival analysis with only cognitive predictors. 2st.cov and Jnt.cov are the corresponding analyses with additional predictors (T1age, smoking status, and self-reported health). re.LC = random effect for linear change. Start Age = age in years (offset from 65) at study induction. HR (scaled) is the hazard ratio for a + 1SD difference in the corresponding predictor. For smoker, HR is just the effect of smoking vs. not smoking. Brackets denote 95% CI. ^a^95% CI for Smoker and SRH are not shown due to space considerations; None of these overlapped zero.*

For further comparison, we calculated hazard ratios for 1SD differences in the cognitive predictors.^3^ For Gc, linear changes more strongly predicted mortality risk in unadjusted models, whereas Gc intercepts more strongly predicted mortality risk in adjusted models. For Gf, linear changes more strongly predicted mortality risk than did intercepts across analyses. With respect to other covariates, age at study inception was negatively associated with mortality risk—likely a selection effect wherein being older at study inception indicates increased survivability (Lindenberger et al., 2002). Smoking predicted higher mortality risk, and better SRH predicted lower mortality risk. Scaled hazard ratios for non-cognitive predictors differed little across two-stage and joint analyses.

## Discussion

We compared two-stage vs. joint longitudinal-survival estimation approaches using data from a large sample study of middle-aged and older participants. Differences in estimated cognitive declines across standalone MLM vs. joint modeling frameworks were only evident at far ends of the age spectrum, and these differences were negligible after adjustment for differences in smoking and self-rated health (SRH). In contrast, cognition-mortality associations were conspicuously more pronounced when estimated using joint models vs. using a two-stage procedure. Importantly, longitudinal associations between crystallized intelligence and mortality risk emerged only when estimated jointly.

### Cognitive Trajectories in Joint vs. Standalone Analyses

Compared to estimates from standalone multilevel models, cognitive trajectories estimated jointly with mortality risk showed sharper declines at ends of the age spectrum where participant data were fewer; however, these differences were negligible after adjustment for age at study entry, smoking, and SRH (Figures 1, 2), variables that in previous analyses we identified as comparatively strongly related to mortality risk in this participant sample. This outcome suggests that for research primarily focused on modeling longitudinal changes in cognitive performance, the use of standalone MLM with inclusion of appropriate health- and survival-related covariates may suffice for common use cases.

We could only identify two prior reports of cognitive declines across both two-stage and joint longitudinal-survival statistical frameworks. In these studies, memory and visuospatial reasoning showed non-significant declines in both frameworks (Muniz-Terrera et al., 2011, 2018). This differed from our results wherein performance in Gc and Gf declined (approximately −0.1 *SD*/decade and −0.6 *SD*/decade, respectively) in both frameworks. These differences may be because the studies estimated changes in different cognitive abilities or because of different sample selection criteria, including differences in sample size (and by extension, statistical power necessary to estimate reliable change).

We also examined the selection effects of excluding survivors (approximately 25% of participants) on cognitive trajectories estimated in standalone MLM. We found that decedents on average performed worse (lower baseline performance) than participants generally, which is consistent with previous studies (e.g., Bosworth et al., 1999; Small et al., 2003). However, these differences were greatly reduced in models adjusted for differences in starting age, smoking, and self-rated health. This is consistent with prior findings that adjustment for health-related covariates partially remedies the effects of death-related dropout on estimated performance (Rabbitt et al., 2005). Taken together, these results point to the importance of including survival-related covariates in models of adult developmental cognitive change estimated independently of mortality risk.

### Cognition-Survival Associations in Joint vs. Two-Stage Methodologies

Gc was significantly associated with mortality risk only in joint analyses, whereas associations between Gf and mortality risk were robust across both analytical frameworks. Scaled hazard ratios (i.e., risk reduction per + 1SD better cognitive performance) were 10–29% more pronounced when estimated using a joint vs. two-stage procedure. These outcomes were in general the result both of increased predictive strength and improved accuracy (narrower 95% CI) in cognition-mortality associations modeled jointly vs. using a two-stage procedure. These results align with those from the only other study we know of to report cognition-mortality associations for both two-stage and joint procedures, which showed that cognition-mortality associations were significant only in joint models (Ghisletta et al., 2006). However, in this earlier study, cognitive abilities were examined simultaneously in two-stage analyses but independently in joint analyses. Here, cognitive abilities were examined singularly across both methodologies. Thus, to our knowledge, this is the first study allowing for direct comparison of cognition-mortality associations across two-stage and joint longitudinal-survival frameworks.

That Gc was only significantly predictive of mortality risk in joint models^4^ may have important substantive implications. White and Cunningham (1988) asserted that, compared to Gf, Gc would be less affected by aging and more markedly affected by end-of-life processes. To date there has been little support for this hypothesis (Bäckman and MacDonald, 2006), but the current results indicate that the longitudinal association between Gc and mortality risk may be underestimated when not modeled jointly (i.e., when mortality-related dropout remains unaccounted for). This is probably due to the increased statistical efficiency (Rizopoulos, 2012, 2016) of joint models, where missingness in longitudinal scores is directly accounted for by observed differences in mortality risk, and missingness in mortality information (right censored time-of-death for survivors) is accounted for by observed differences in longitudinal performance. Additionally, the growing literature on cognitive reserve (Stern, 2009) suggests that well developed crystallized abilities (e.g., verbal intelligence, lifestyle skills, cultural and relational learning) may buffer against the deleterious impacts of neurodegenerative diseases. Just how well-preserved such crystallized abilities are in later life, and by extension their utility as compensatory factors for protecting against declines in abilities more prone to neurodegenerative conditions, may possibly be overestimated when not conditioned on differences in mortality risk. This point bears consideration for future research in this domain.

### Limitations

In joint models, longitudinal parameter estimates were adjusted for death-related dropout. However, study attrition can occur for other reasons: E.g., moving for a new job or retirement, or due to mobility problems unrelated to more serious health issues. We were not able to conduct a more extensive analysis of dropout-related missingness as we lacked information about participants’ reasons for leaving. However, prior work has identified value in modeling other sources of informative dropout for estimating cognitive decline (e.g., Sliwinski et al., 2003). We encourage future researchers to consider such variables in addition to mortality risk. Further, we did not consider differences in cognitive declines with respect to specific cause of death, notably related to dementia, because these data were not available at the 2012 MLSC mortality census, which we used here. Dementia-related deaths reported at an earlier 2008 census constituted approximately 3% of all deaths in this sample (well beneath the U.K. population average in persons over 65 years of age).

On a technical level, at the time of analysis, JMBayes required output from standalone multilevel models estimated with maximum likelihood (ML) to obtain starting values for MCMC estimation. This approach encouraged us to test for the best model parameterizations (e.g., intercept-only vs. intercept plus linear slope models) in a standalone MLM framework. In hindsight, it would have been preferable to re-test model parameterization within the joint longitudinal-survival framework (a) given that we observed some relative improvements in the accuracy of longitudinal parameters under joint estimation, (b) because a simpler subset of longitudinal parameters may have been preferred after conditioning on survival status, and (c) because model comparison under ML differs from that under MCMC.

Although our primary substantive focus was in the area of cognitive epidemiology (i.e., cognition as predictive of mortality risk), we also compared effects on cognitive trajectories when estimated in standalone MLM vs. when estimated conditionally on mortality risk. It has been argued that these group-average trajectories pertain to “immortal cohorts” and should be interpreted accordingly. A more recently developed joint modeling approach (Li and Su, 2018) now appears to allow for closed-form estimation of longitudinal changes valid for inferences to mortal (i.e., non-decedent) populations. We look forward to using this approach in future research.

## Conclusion

Cognition-mortality associations were more prominent when estimated with joint longitudinal-survival models vs. using a two-stage estimation procedure. This may have particular significance for detecting associations between crystallized abilities (e.g., verbal skills) and mortality-related processes. For applications wherein only the longitudinal component (e.g., cognitive change) is of substantive interest, inclusion of survival-related covariates in standalone multilevel models may provide estimates of declines similar to those obtained from a joint modeling approach.

## Data Availability Statement

The raw data supporting the conclusions of this article will be made available by the authors, without undue reservation.

## Ethics Statement

The studies involving human participants were reviewed and approved by the Department of Psychology Internal Ethics Committee, University of Manchester; the University of Manchester Ethics Committee; the Greater Manchester National Institute for Health Trust Ethics Committee. The patients/participants provided their written informed consent to participate in this study.

## Author Contributions

SA performed all analyses and was responsible for manuscript preparation. SC provided statistical guidance. PR was responsible for data collection and provided editorial feedback during manuscript preparation. PG assisted with statistical interpretation, provided editorial feedback during manuscript preparation, and was responsible for general oversight. All authors contributed to the article and approved the submitted version.

## Conflict of Interest

The authors declare that the research was conducted in the absence of any commercial or financial relationships that could be construed as a potential conflict of interest.

## Publisher’s Note

All claims expressed in this article are solely those of the authors and do not necessarily represent those of their affiliated organizations, or those of the publisher, the editors and the reviewers. Any product that may be evaluated in this article, or claim that may be made by its manufacturer, is not guaranteed or endorsed by the publisher.

## Footnotes

1 See Rizopoulos (2012, p. 89–92) for additional technical information on how joint models accommodate repeated observations “missing not at random,” or MNAR.

2 Retest effects were nearly always non-significant when starting age was included as a covariate. Retest was also not a predictor in survival models. For these reasons, we chose to exclude retest as a covariate.

3 Percent reductions in mortality risk were calculated as (1 − HR_Zp_) ^∗^ 100, where HR_Zp_ is the hazard ratio corresponding to + 1SD difference in predictor p (Zp). Zp was calculated as the square root of the estimated random variance for the given predictor. Thus, for women’s intercept for fluid intelligence (in the joint model, with covariates): HR_Zp_ = 1 −exp(−0.012 ^∗^ √52.78) = 1 −0.916, or 8.4% lower risk per + 1SD difference.

4 We previously showed that intercept for Gc was negatively associated with mortality risk in MLSC participants using a two-stage procedure (Aichele et al., 2015). Mortality information for the current study was updated to a more recent census and thus provided more information on decedents. This may explain why the association between Gc intercept and mortality risk was not observed in the current two-stage procedures.

## Supplementary Material

Supplementary Data Sheet 1 JM Bayes example script.

Supplementary Data Sheet 2 Simulated data to run with JM Bayes example script.

Supplementary Data Sheet 3 Model fit comparisons summary statistics.
