# Supplementary material for: Cognition-Mortality Associations Are More Pronounced When Estimated Jointly in Longitudinal and Time-to-Event Models
Source: Front Psychol. 2021 Aug 6;12:708361. doi: 10.3389/fpsyg.2021.708361 (PMC8378533; doi:10.3389/fpsyg.2021.708361)
Supplement: Supplementary Data Sheet 1 — JM Bayes example script. [file Data_Sheet_1.pdf]

```r
# ------------------------------------------------------------------------------
# Sample code to show use of JMbayes for joint longitudinal-survival modeling
# - as applied to simulated data (note, this runs as intendend but does not
# provide convincing results due to limitations inherent to the simulated data)
# ------------------------------------------------------------------------------

# clear memory
rm(list=ls())

# load libraries
require(nlme)
require(survival)
require('JMbayes')

# load data
wd = "/path/to/your/data/directory/"
d = read.csv(paste(wd, 'S2_JMB_example_data.csv', sep=''), header = TRUE)
str(d)

# Use age at testing, centered, as longitudinal time metric
d$Time = d$AgeTest - median(d$AgeTest)

# wide-format data for survival model (important that same IDs are present
# in long-format and wide format data, otherwise joint models won't work)
dW = unique(d[,c(1:7)])


# ------------------------------------------------------------------------------
# Multilevel change models
#     note that lme() uses REML estimation by default, which is preferred for
#     parameter estimates but not appropriate for model testing. For the latter,
#     models need to be fit with ML.
# ------------------------------------------------------------------------------

# intercept-only model
mlm_00 = lme(fixed = GF ~ 1 + AgeStart, random = ~ 1 | ID,
             data = d,
             method = "ML"
             )

# + linear slope (fixed and random effects)
mlm_01 = lme(fixed = GF ~ 1 + Time + AgeStart, random = ~ 1 + Time | ID,
             data = d,
             method = "ML"
             )

# which is the better fit?
anova(mlm_00, mlm_01)

# mlm_01 has better fit, so re-estimate with REML for param ests
mlm_01 = update(mlm_01, method = "REML")
summary(mlm_01)


# ------------------------------------------------------------------------------
# Cox proportional hazards survival model
# ------------------------------------------------------------------------------

srv_00 = Surv(dW$AgeLastObs, dW$Dead)
srv_01 = coxph(srv_00 ~ 1 + AgeStart + Female + Smoker,
               data = dW,
               x = TRUE,
               model = TRUE
               )
summary(srv_01)
```

```r

# ------------------------------------------------------------------------------
# Joint longitudinal-survival model with shared random effects parameterization
# ------------------------------------------------------------------------------

jnt_01 = jointModelBayes(lmeObject  = mlm_01,
                         survObject = srv_01,
                         timeVar = "Time",
                         param = "shared-RE",
                         n.iter = 5000,       # MCMC iterations, adjust higher
                         n.burnin = 500       # MCMC burn-in, adjust higher
                         )
summary(jnt_01)
```
