# Supplementary material for: Cognition-Mortality Associations Are More Pronounced When Estimated Jointly in Longitudinal and Time-to-Event Models
Source: Front Psychol. 2021 Aug 6;12:708361. doi: 10.3389/fpsyg.2021.708361 (PMC8378533; doi:10.3389/fpsyg.2021.708361)
Supplement: Supplementary Data Sheet 3 — Model fit comparisons summary statistics. [file Data_Sheet_3.pdf]

## S3. Longitudinal Model Comparisons

### Intercept (m1) vs. Linear slope (m2)

| Model | df | AIC | BIC | logLik | L.Ratio | p-value |
|---|---|---|---|---|---|---|
| *Gc, Females (All), Chronological Age* | | | | | | |
| m1 | 3 | 48428.37 | 48449.75 | -24211.18 | | |
| m2 | 6 | 48158.81 | 48201.57 | -24073.40 | 275.5588 | <.0001 |
| *Gc, Females (Decedents), Chronological Age* | | | | | | |
| m1 | 3 | 32806.97 | 32827.11 | -16400.48 | | |
| m2 | 6 | 32652.47 | 32692.76 | -16320.24 | 160.4953 | <.0001 |
| *Gc, Males (All), Chronological Age* | | | | | | |
| m1 | 3 | 19960.54 | 19979.23 | -9977.268 | | |
| m2 | 6 | 19815.61 | 19853.00 | -9901.806 | 150.9234 | <.0001 |
| *Gc, Males (Decedents), Chronological Age* | | | | | | |
| m1 | 3 | 16420.47 | 16438.52 | -8207.233 | | |
| m2 | 6 | 16298.62 | 16334.74 | -8143.311 | 127.844 | <.0001 |
| *Gf, Females (All), Chronological Age* | | | | | | |
| m1 | 3 | 51467.69 | 51489.07 | -25730.85 | | |
| m2 | 6 | 50170.58 | 50213.34 | -25079.29 | 1303.114 | <.0001 |
| *Gf, Females (Decedents), Chronological Age* | | | | | | |
| m1 | 3 | 34608.20 | 34628.34 | -17301.10 | | |
| m2 | 6 | 33610.52 | 33650.81 | -16799.26 | 1003.671 | <.0001 |
| *Gf, Males (All), Chronological Age* | | | | | | |
| m1 | 3 | 21659.05 | 21677.74 | -10826.52 | | |
| m2 | 6 | 20993.21 | 21030.59 | -10490.60 | 671.8431 | <.0001 |
| *Gf, Males (Decedents), Chronological Age* | | | | | | |
| m1 | 3 | 17721.79 | 17739.85 | -8857.897 | | |
| m2 | 6 | 17105.89 | 17142.00 | -8546.946 | 621.9018 | <.0001 |

## Linear slope (m1) vs. Quadratic slope (m2)

| Model | df | AIC | BIC | logLik | L.Ratio | p-value |
|---|---|---|---|---|---|---|
| *Gc, Females (All), Chronological Age* | | | | | | |
| m1 | 6 | 48158.81 | 48201.57 | -24073.40 | | |
| m2 | 7 | 48077.08 | 48126.97 | -24031.54 | 83.72873 | <.0001 |
| *Gc, Females (Decedents), Chronological Age* | | | | | | |
| m1 | 6 | 32652.47 | 32692.76 | -16320.24 | | |
| m2 | 7 | 32611.16 | 32658.16 | -16298.58 | 43.31456 | <.0001 |
| *Gc, Males (All), Chronological Age* | | | | | | |
| m1 | 6 | 19815.61 | 19853.0 | -9901.806 | | |
| m2 | 7 | 19762.08 | 19805.7 | -9874.039 | 55.53427 | <.0001 |
| *Gc, Males (Decedents), Chronological Age* | | | | | | |
| m1 | 6 | 16298.62 | 16334.74 | -8143.311 | | |
| m2 | 7 | 16262.15 | 16304.29 | -8124.075 | 38.47131 | <.0001 |
| *Gf, Females (All), Chronological Age* | | | | | | |
| m1 | 6 | 50170.58 | 50213.34 | -25079.29 | | |
| m2 | 7 | 49380.55 | 49430.43 | -24683.27 | 792.0317 | <.0001 |
| *Gf, Females (Decedents), Chronological Age* | | | | | | |
| m1 | 6 | 33610.52 | 33650.81 | -16799.26 | | |
| m2 | 7 | 33226.78 | 33273.78 | -16606.39 | 385.7421 | <.0001 |
| *Gf, Males (All), Chronological Age* | | | | | | |
| m1 | 6 | 20993.21 | 21030.59 | -10490.60 | | |
| m2 | 7 | 20698.04 | 20741.66 | -10342.02 | 297.1654 | <.0001 |
| *Gf, Males (Decedents), Chronological Age* | | | | | | |
| m1 | 6 | 17105.89 | 17142.00 | -8546.946 | | |
| m2 | 7 | 16918.23 | 16960.36 | -8452.115 | 189.6632 | <.0001 |

## QS w/out T1 time (m1) vs. QS w/T1time & T1time*LS (m2)

| Model | df | AIC | BIC | logLik | L.Ratio | p-value |
|---|---|---|---|---|---|---|
| *Gc, Females (All), Chronological Age* | | | | | | |
| m1 | 7 | 48077.08 | 48126.97 | -24031.54 | | |
| m2 | 9 | 47785.95 | 47850.09 | -23883.97 | 295.1299 | <.0001 |
| *Gc, Females (Decedents), Chronological Age* | | | | | | |
| m1 | 7 | 32611.16 | 32658.16 | -16298.58 | | |
| m2 | 9 | 32422.35 | 32482.77 | -16202.17 | 192.8122 | <.0001 |
| *Gc, Males (All), Chronological Age* | | | | | | |
| m1 | 7 | 19762.08 | 19805.70 | -9874.039 | | |
| m2 | 9 | 19616.86 | 19672.94 | -9799.430 | 149.2182 | <.0001 |
| *Gc, Males (Decedents), Chronological Age* | | | | | | |
| m1 | 7 | 16262.15 | 16304.29 | -8124.075 | | |
| m2 | 9 | 16106.62 | 16160.79 | -8044.310 | 159.531 | <.0001 |
| *Gf, Females (All), Chronological Age* | | | | | | |
| m1 | 7 | 49380.55 | 49430.43 | -24683.27 | | |
| m2 | 9 | 47829.04 | 47893.18 | -23905.52 | 1555.503 | <.0001 |
| *Gf, Females (Decedents), Chronological Age* | | | | | | |
| m1 | 7 | 33226.78 | 33273.78 | -16606.39 | | |
| m2 | 9 | 32419.48 | 32479.91 | -16200.74 | 811.2993 | <.0001 |
| *Gf, Males (All), Chronological Age* | | | | | | |
| m1 | 7 | 20698.04 | 20741.66 | -10342.02 | | |
| m2 | 9 | 20114.97 | 20171.05 | -10048.49 | 587.0683 | <.0001 |
| *Gf, Males (Decedents), Chronological Age* | | | | | | |
| m1 | 7 | 16918.23 | 16960.36 | -8452.115 | | |
| m2 | 9 | 16507.67 | 16561.83 | -8244.833 | 414.5641 | <.0001 |

## QS + T1 time w/Retest (m1) vs. QS + T1 time w/out Retest (m2)

| Model | df | AIC | BIC | logLik | L.Ratio | p-value |
|---|---|---|---|---|---|---|
| *Gc, Females (All), Chronological Age* | | | | | | |
| m1 | 9 | 47785.95 | 47850.09 | -23883.97 | | |
| m2 | 10 | 47785.19 | 47856.46 | -23882.59 | 2.762048 | 0.0965 |
| *Gc, Females (Decedents), Chronological Age* | | | | | | |
| m1 | 9 | 32422.35 | 32482.77 | -16202.17 | | |
| m2 | 10 | 32421.44 | 32488.58 | -16200.72 | 2.909795 | 0.088 |
| *Gc, Males (All), Chronological Age* | | | | | | |
| m1 | 9 | 19616.86 | 19672.94 | -9799.430 | | |
| m2 | 10 | 19615.99 | 19678.31 | -9797.996 | 2.868212 | 0.0903 |
| *Gc, Males (Decedents), Chronological Age* | | | | | | |
| m1 | 9 | 16106.62 | 16160.79 | -8044.310 | | |
| m2 | 10 | 16107.76 | 16167.95 | -8043.881 | 0.8584507 | 0.3542 |
| *Gf, Females (All), Chronological Age* | | | | | | |
| m1 | 9 | 47829.04 | 47893.18 | -23905.52 | | |
| m2 | 10 | 47826.75 | 47898.02 | -23903.38 | 4.294747 | 0.0382 |
| *Gf, Females (Decedents), Chronological Age* | | | | | | |
| m1 | 9 | 32419.48 | 32479.91 | -16200.74 | | |
| m2 | 10 | 32421.36 | 32488.50 | -16200.68 | 0.1193403 | 0.7298 |
| *Gf, Males (All), Chronological Age* | | | | | | |
| m1 | 9 | 20114.97 | 20171.05 | -10048.49 | | |
| m2 | 10 | 20105.23 | 20167.54 | -10042.62 | 11.73956 | 6e-04 |
| *Gf, Males (Decedents), Chronological Age* | | | | | | |
| m1 | 9 | 16507.67 | 16561.83 | -8244.833 | | |
| m2 | 10 | 16496.94 | 16557.12 | -8238.468 | 12.72902 | 4e-04 |
